# Implementation of Occupied and Virtual Edmiston–Ruedenberg Orbitals Using Cholesky Decomposed Integrals

**DOI:** 10.1021/acs.jctc.2c00261

**Published:** 2022-07-20

**Authors:** Sarai Dery Folkestad, Regina Matveeva, Ida-Marie Høyvik, Henrik Koch

**Affiliations:** Department of Chemistry, The Norwegian University of Science and Technology, Trondheim 7491, Norway

## Abstract

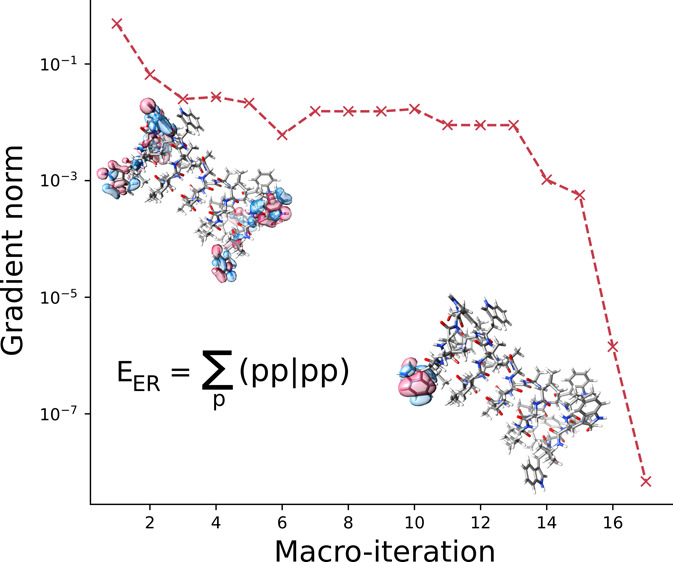

We present a trust-region optimization of the Edmiston–Ruedenberg orbital localization function. The approach is used to localize both the occupied and the virtual orbitals and is the first demonstration of general virtual orbital localization using the Edmiston–Ruedenberg localization function. In the Edmiston–Ruedenberg approach, the sum of the orbital self-repulsion energies is maximized to obtain the localized orbitals. The Cholesky decomposition reduces the cost of transforming the electron repulsion integrals, and the overall scaling of our implementation is . The optimization is performed with all quantities in the molecular orbital basis, and the localization of the occupied orbitals is often less expensive than the corresponding self-consistent field (SCF) optimization. Furthermore, the occupied orbital localization scales linearly with the basis set. For the virtual space, the cost is significantly higher than the SCF optimization. The orbital spreads of the resulting virtual Edmiston–Ruedenberg orbitals are larger than for other, less expensive, orbital localization functions. This indicates that other localization procedures are more suitable for applications such as local post-Hartree–Fock calculations.

## Introduction

The idea of orbital localization through maximizing the orbital–orbital self-repulsion energy dates back to the early 1950s, with the work of Lennard-Jones and Pople.^1^ They considered the benefits of using symmetry equivalent (rather than canonical) Hartree–Fock orbitals to gain understanding of molecular structure and remarked that such orbitals reduce the exchange contribution to the energy and consequently increase the orbital self-repulsion energy. These energy conditions were first explicitly used to localize occupied orbitals by Edmiston and Ruedenberg.^2,3^ They suggested that the maximum of the orbital self-repulsion energy could be found either through a gradient based (steepest ascent) approach or through the use of Jacobi sweeps—an optimization procedure that iteratively rotates pairs of orbitals until the specified energy function converges to a stationary point.

Conjugate gradient approaches and the Broyden–Fletcher–Goldfarb–Shanno (BFGS) algorithm have also been applied to the Edmiston–Rudenberg localization problem.^4−6^ Leonard and Luken^7^ suggested a mixed Newton–Raphson/quasi-Newton approach, where they approximated the Hessian matrix by its diagonal elements and alternately used the approximated and the full Hessian to obtain quadratic convergence; although the approach was formulated for the Edmiston–Ruedenberg localization function, it was only applied to Foster–Boys^2,8,9^ localization, due to the cost to compute the electron repulsion integrals needed for the Hessian. In the Foster–Boys approach, the electron repulsion integrals are replaced by products of dipole integrals, yielding the same form of the gradient and Hessian as obtained for the Edmiston–Ruedenberg localization function.

Gradient based approaches and Jacobi sweeps are inefficient for larger systems and particularly incapable of localizing the virtual space.^10−12^ The latter is also the case for Newton–Raphson or quasi-Newton solvers.^11^ In contrast to occupied orbital localization, saddle points and close lying local minima can be encountered in virtual orbital localization.^13−15^ These features are common to all localization functions, including the Edmiston–Ruedenberg approach, and severely complicate the optimization problem. For any initial guess of the localized virtual orbitals, the Hessian will have several large negative eigenvalues. The approaches mentioned above will in general not be able to converge when the initial Hessian is not positive definite.

The development of local correlation methods^16−21^ and correlated active space approaches that rely on localized orbitals^22−25^ has motivated the development of robust solvers for the orbital localization problem for both the occupied and virtual orbitals. Jansík et al.^26^ and Høyvik et al.^27^ adapted Fletcher’s trust-region minimization method^28^ and applied it successfully to localization functions for both sets of orbitals. The trust-region approach is based on defining a local region, in which the objective function is well represented by a quadratic approximation. At each step of the optimization, a minimum of the approximated function is found. The distinctive feature of this method is the quadratic convergence of the localization function even if the initial Hessian matrix has negative eigenvalues.^29^ The trust-region optimization has been applied to localization by minimizing powers of the orbital spread (a special case of which is Foster–Boys localization),^26^ to minimization of the fourth central moment,^30^ and for the Pipek–Mezey localization function.^31^ It has also been demonstrated to work for basis sets augmented with diffuse basis functions.^32^

The popularity of the Edmiston–Ruedenberg localization function is limited compared to that of Pipek–Mezey^33^ and Foster–Boys^2,8,9^ localization. This is likely due to the prohibitive  scaling of a naive implementation: the electron repulsion integrals must be transformed to the updated molecular orbital (MO) basis in every iteration. Significant progress toward minimizing the scaling of the Edmiston–Ruedenberg localization procedure for the occupied orbitals was made by Subotnik et al.^13,14^ Subotnik et al. first introduced a purely gradient based approach^13^ using a DIIS (direct inversion of the iterative subspace)^34^ accelerated algorithm. Although the integral manipulations are linear scaling, the overall asymptotic scaling is , due to dense linear algebra operations and memory manipulations. They exploited both the locality of the atomic orbitals (AOs) and of their initial guess for the Edmiston–Ruedenberg orbitals (which was taken to be Foster–Boys orbitals) to screen the integrals. The method was subsequently improved, reducing the prefactor of the  integral manipulations using the resolution-of-identity^35^ approximation and eliminating convergence issues of the gradient-only approach by including information from the Hessian. For the determination of the lowest eigenvalue of the Hessian, the Davidson algorithm^36^ was applied, and in the case of a saddle point (negative Hessian eigenvalue), the prescription of Seeger and Pople^37^ was followed. However, the additional computational costs needed for linear transformations by the Hessian were not reported. Naively, this transformation relies on the computation of  electron repulsion integrals.^7^ Subotnik et al. only considered the occupied orbitals. To our knowledge, no attempts have been made to localize virtual orbitals using the Edmiston–Ruedenberg approach.

In this paper, we present a trust-region approach to determine Edmiston–Ruedenberg orbitals for both the occupied and virtual space. The trust-region optimization ensures convergence to a minimum of the Edmiston–Ruedenberg localization function, and Cholesky decomposition of the electron repulsion integrals^38^ is used to reduce the cost of integral evaluation and transformation. Performance of the approach, in terms of convergence properties and computational cost, is considered, and so is the effect of integral approximation on the set of localized orbitals.

## Theory

The Hartree–Fock energy and density are invariant to rotations among the occupied orbitals and among the virtual orbitals, separately. Edmiston–Ruedenberg orbitals^2^ are identified by maximizing the orbital self-repulsion energy
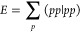
1where the summation is over either the occupied or virtual orbitals. This is equivalent to minimizing the exchange contribution to the energy
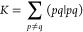
2and the interorbital repulsion energy
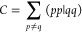
3

In practice, we will consider a minimization of *f* = −*E* given in eq 1, since the trust-region solver is implemented for minimizations. A minimum is obtained by successive rotations among the orbitals under consideration (occupied or virtual)
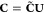
4Here, **U** is an orthogonal matrix, parametrized as the exponential of some real-valued antisymmetric matrix **κ**:
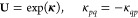
5This parametrization ensures that the orthonormality of the orbitals is preserved at all times. By inserting eq 5 into the Edmiston–Ruedenberg functional, we get
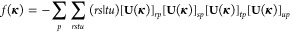
6Inserting the Taylor expansion of **U**(**κ**) = exp(**κ**) about zero
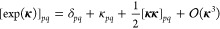
7into the expression for the orbital self-repulsion energy given in eq 6 and organizing the terms in equal orders of **κ**, we obtain
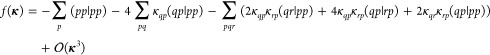
8Using the relation
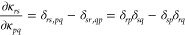
9we find that the first derivative of *E*(**κ**) with respect to κ_*pq*_ is given by
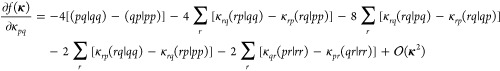
10We can identify the gradient (**f**^[1]^) as the terms that are zeroth order in **κ** and the linear transformation by the Hessian (**f**^[2]^**κ**) as the terms linear in **κ**
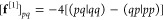
11
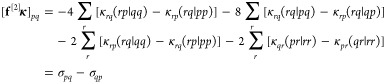
12where
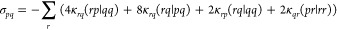
13The diagonal elements of the Hessian are used as a preconditioner and can be retrieved from the linear transformation by letting, for each index *pq*, κ_*rs*_ = δ_*rs*,*pq*_. We obtain
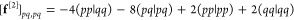
14

### Energy Optimization Procedure

We now describe the most relevant characteristics of the trust-region method used to localize the orbitals in this work. For a more detailed description, we refer the reader to ref (27). The trust-region method^28^ is based on approximating an objective function (here the orbital self-repulsion energy given in eq 8) by a second-order Taylor expansion and identifying the region in which this expansion is a good representation of the objective function.^11^ This method is also called the restricted step method due to the limitation of the step by the region of validity of the Taylor series. We consider a second-order Taylor series of an energy functional *f*(κ) expanded around **κ** = **0**
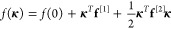
15where **f**^[1]^ and **f**^[2]^ are the gradient and Hessian, respectively, evaluated at the expansion point
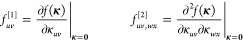
16Differentiating eq 15 with respect to **κ** and setting the resulting equation equal to zero yields the Newton step
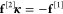
17This step is accepted if the Hessian is positive definite.

If the Hessian is not positive definite, a step which gives the minimum value of *f*(**κ**) on the boundary of the trust-region (|**κ**| = *h*) has to be determined. For this reason, a Lagrangian is defined
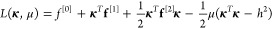
18where μ is a Lagrange multiplier and |**κ**| is taken to be the *l*_2_-norm of **κ**. The stationary points of eq 18 are given by
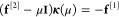
19
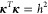
20Equation 19 is a level-shifted Newton equation which has several solutions **κ**. However, restricting μ to be lower than the lowest eigenvalue of the Hessian **f**^[2]^ results in a **κ** that represents the minimum.

In order to find the minimizing solution of eq 19, an augmented Hessian eigenvalue equation is introduced:^27^
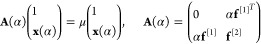
21It has two parts
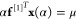
22
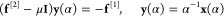
23the latter of which is the level-shifted Newton equation (equal to eq 19). By the Hylleraas–Undheim–MacDonald^39,40^ theorem, the lowest eigenvalue μ of eq 21 is guaranteed to be lower than the lowest eigenvalue of **f**^[2]^. Hence, if **f**^[2]^ has negative eigenvalues, solving eq 21 for the lowest eigenvalue is equivalent to solving eq 19 for a positive definite (**f**^[2]^ – μ**I**). Furthermore, there exists some α for which the eigenvector corresponding to the lowest eigenvalue satisfies |**κ**| = |α^–1^**x**(α)| ≈ *h*.

The trust-region optimization for orbital localization is performed in the following steps:

1. For the current set of orbitals, the function value (*f*) and the gradient (**f**^[1]^) are computed. If the gradient norm is smaller than the convergence threshold, the optimization is done; otherwise, we continue to step 2.
2. Equation 21 (or eq 17, if the Hessian is positive definite) is solved to find a set of orbital rotation parameters. Equation 21 is solved iteratively, using a reduced space algorithm adapted from the Davidson procedure.^36^ A detailed description of how this is done can be found in ref (27) or (41). The Davidson iterations are called the microiterations of the trust-region procedure.
3. Once the orbital rotation parameters **κ** are obtained, a line search is performed. The trust radius, *h*, is chosen to be no larger than  to ensure that the quadratic approximation of *f* is a good approximation. However, since the *l*_2_-norm is extensive, this can lead to excessively small steps, especially for larger systems and far away from the minimum. Following the microiterations, we have identified the **κ** that minimizes the quadratic function within the trust-region. It is then a simple and pragmatic approach to perform the line search by applying exp(*n**κ***) for some integer *n* that minimizes the energy. The line search is implemented by applying the transformation given by eqs 4 and 5 several times, each time confirming that the function value decreases. That is,
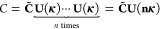
24
4. The trust radius is updated as suggested in ref (42), by considering the ratio of the approximated change in *f* (from the quadratic approximation) to the actual change in *f*. Steps 1–4 form a macroiteration of the trust-region procedure. We return to step 1.

### Edmiston–Ruedenberg Orbitals Using Cholesky Decomposed Electron Repulsion Integrals

In each iteration of the Edmiston–Ruedenberg localization procedure, the electron repulsion integral matrix must be transformed to the updated MO basis. In order to avoid the  scaling of this procedure, we introduce the Cholesky factorization of the integral matrix^38^
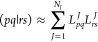
25where the number of Cholesky vectors *N*_*J*_ scales linearly with the system size.^43^ The accuracy of this factorization is controlled through the threshold, τ, used in the decomposition. It has been demonstrated that, with an efficient implementation, this decomposition is often less expensive than the SCF optimization.^44,45^

In each macroiteration, the Cholesky vectors must be transformed to the new basis
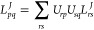
26a process which is  scaling, where *n* is a measure of the size of the orbital space that is optimized and *N* is proportional to the number of AOs. Inserting the Cholesky factorization in eq 25 into the expression for the Edmiston–Ruedenberg functional (eq 1), we get
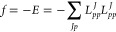
27which scales as . Similarly for the gradient, Hessian transformation, and the diagonal Hessian, we get
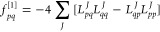
28
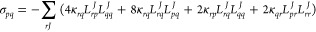
29
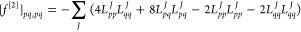
30which entail costs that scale as , , and , respectively. Therefore, the overall iterative cost of the Edmiston–Ruedenberg procedure using the Cholesky vectors is .

To compute the Edmiston–Ruedenberg energy, gradient, and Hessian transformation, we introduce the following intermediates
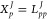
31
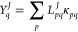
32
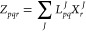
33
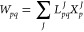
34Intermediates **Y** and **W** have an  cost. The **Z** intermediate, together with the basis transformation of the Cholesky vectors, has an  cost. In our implementation, **L** is transformed and **Z** is constructed only once per macroiteration, and they are stored in memory. Alternatively, they can be stored on disk and be read in batches to reduce memory usage. The remaining intermediates are constructed on the fly (as needed in each microiteration).

In terms of these intermediates, we get the following expression for the energy, gradient, Hessian transformation, and diagonal Hessian
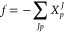
35
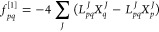
36
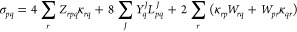
37
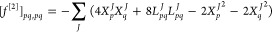
38and the cost of each microiteration (approximately equal to the cost of σ_*pq*_) is then . Note that only MO quantities are used throughout the localization procedure. In particular, for the localization of the occupied orbitals, only the number of Cholesky vectors will increase with increasing basis set (while keeping the system fixed); all other dimensions are unchanged and equal to the number of occupied orbitals.

Each macroiteration **L** and **Z** is computed, and because of their  scaling, these contributions will determine the overall cost for sufficiently large orbital spaces. For smaller orbital spaces, the total time spent in the microiterations can be dominating the cost, i.e., the time to construct the linear transformation by the Hessian, according to eqs 12 and 37; this scales as  but will have a large prefactor due to the large number of microiterations.

### Locality Measure

The (second moment) orbital spread σ_2_^*p*^ can be used to evaluate the locality of an orbital, and it is defined as the square root of the variance of the orbital position
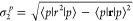
39where  and  give the average position of the orbital.

The orbital spread reflects the spatial extent of an orbital by describing the confinement of an orbital bulk.^11^ A small value of the second moment orbital spread indicates that the orbital bulk is close to the orbital’s average position. However, this value does not necessarily reflect the thickness of the tail, which is also significant for the estimation of the locality of an orbital. For this reason, the fourth moment orbital spread was introduced by Høyvik and Jørgensen^30^
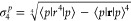
40which is more indicative of the tail’s thickness.

Two measures of the locality of a set of orbitals are the average orbital spread
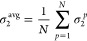
41where *N* is the number of orbitals in the set, and the maximum orbital spread
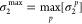
42

Considering either the average orbital spread or the maximum orbital spread will not give a complete picture of the locality of the set of orbitals. The maximum orbital spread reflects the locality of the least local orbital in the set, and hence is an upper bound for the rest of the orbitals. It is a good measure of locality in the sense that it provides a conservative estimate. However, in cases where only a very few orbitals are very delocalized (called outliers^26^), this measure does not accurately represent the locality of the full set. Similar values of the average and the maximum orbital spreads imply that the orbitals in the set are of comparable locality.

## Results

The trust-region solver and the Edmiston–Ruedenberg localization procedure are implemented in a development version of the *e*^*T*^ program,^45^ as described in the previous sections. We use the second moment orbital spread to evaluate the locality of the orbitals, as the integrals required to compute the fourth moment orbital spread are currently not available in *e*^*T*^. All calculations were performed on Intel Platinum 8380 processors, using 80 threads and with up to 2 TB memory available.

A convergence threshold of 10^–7^ is used for the Hartree–Fock equations, and a threshold of 10^–6^ is used for the Edmiston–Ruedenberg orbital localization. The max norm of the gradient is used in both the Hartree–Fock solver and the orbital localization solver. A Cholesky decomposition threshold of τ = 10^–8^ au is considered not to introduce significant approximations in the integrals^44^ and is used unless otherwise stated.

The geometries of arachidic acid, gramicidin, and catenane were obtained from the Supporting Information of refs (5, 46), and (47), respectively. The geometry of circumcoronene was obtained from ref (48) and force field optimized with the Avogadro software package (version 1.2.0)^49^ using the UFF force field. All orbital plots are generated with the UCSF Chimera software package.^50^

### Convergence Properties

**Figure 1:**
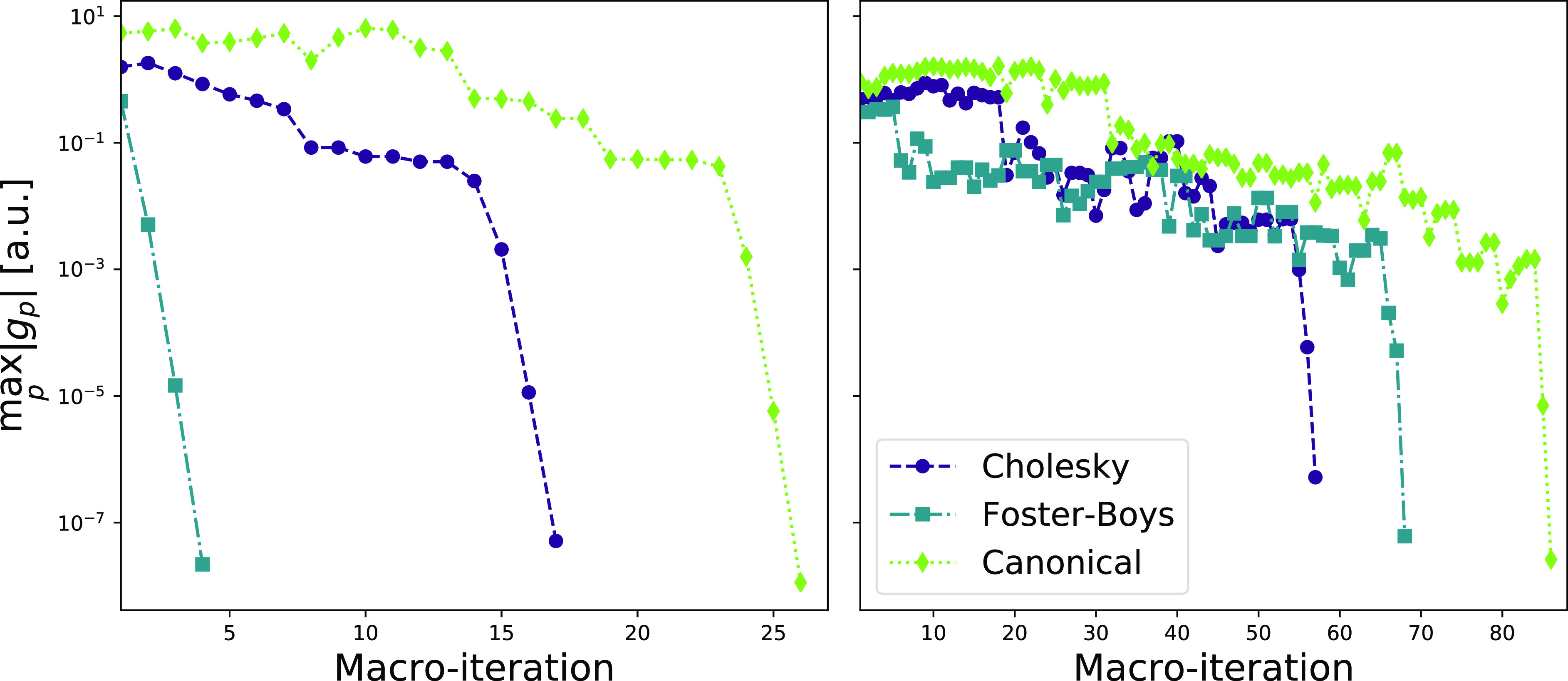
Convergence profiles for the occupied (left panel) and virtual (right panel) orbital localization of arachidic acid in the aug-cc-pVDZ basis using different starting guesses for the Edmiston–Ruedenberg orbitals (canonical, Cholesky, Foster–Boys). The max gradient norm () is used and is given in atomic units (au).

**Figure 2:**
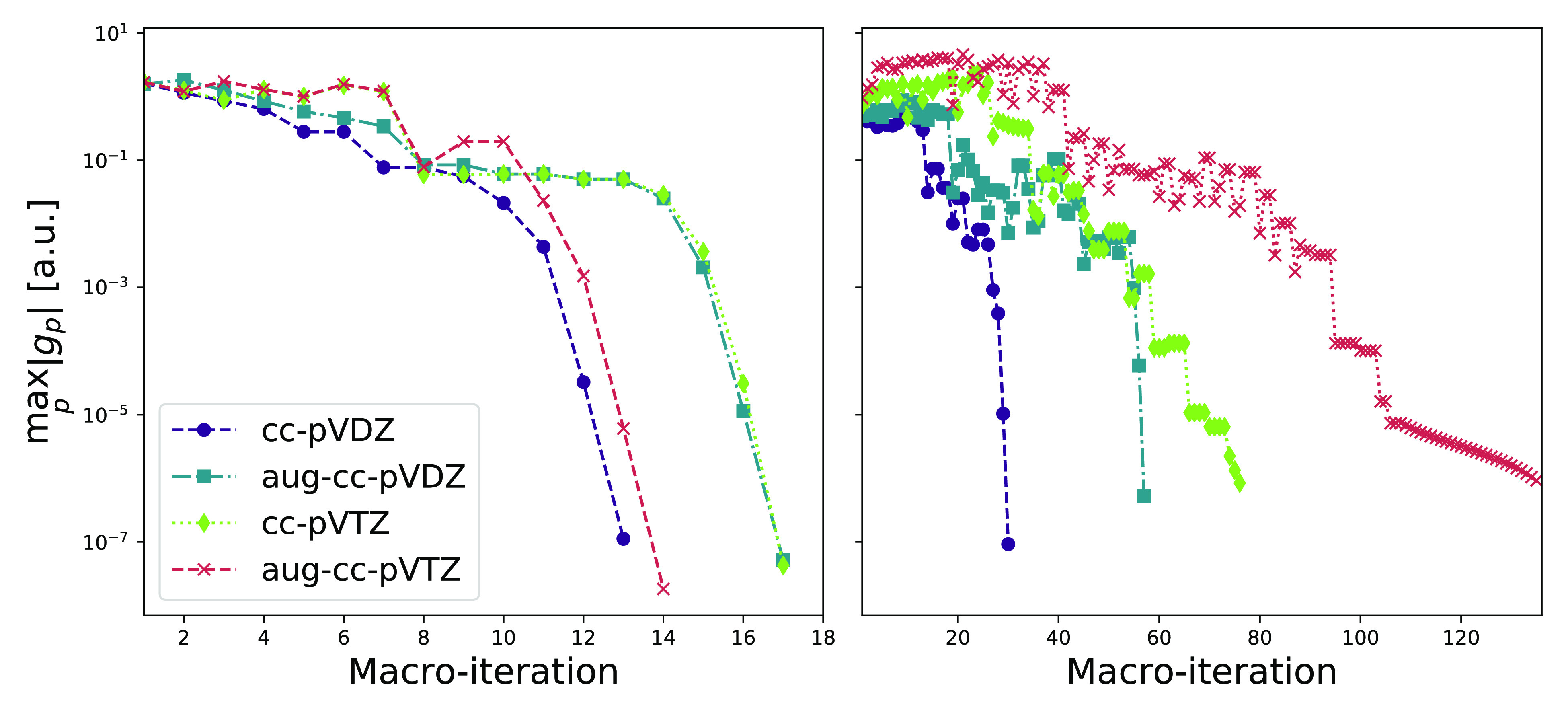
Convergence profiles for the occupied (left panel) and virtual (right panel) orbital localization of arachidic acid. The max gradient norm () is used and is given in atomic units (au).

The convergence profiles for the orbital localization of arachidic acid are given in Figures 1 and 2. In Figure 1, we consider the aug-cc-pVDZ basis and compare the convergence profiles for different initial guesses for the Edmiston–Ruedenberg orbitals, i.e., canonical, Cholesky,^51^ and Foster–Boys orbitals. For the occupied space, a reduction from 26 to 4 macroiterations is achieved with an initial guess of Foster–Boys orbitals, compared to canonical Hartree–Fock orbitals. A Cholesky orbital initial guess resulted in the fastest convergence for the virtual orbitals of arachidic acid. We cannot, in general, conclude that the Cholesky orbitals provide a better starting guess for the virtual Edmiston–Ruedenberg localization. Both Cholesky and Foster–Boys orbitals have negligible costs compared to the Edmiston–Ruedenberg orbitals. However, the Cholesky orbital localization entails a non-iterative  cost, whereas the Foster–Boys orbital localization is iterative .

Orbital localization functions, in particular for virtual orbital localization, are known to have several local minima. The trust-region algorithm only guarantees convergence to a local minimum, and the change in starting guess can often result in convergence to different local minima. For arachidic acid in the aug-cc-pVDZ basis, we do not observe any significant difference in locality resulting from the starting guess.

In Figure 2, we compare the convergence for different basis sets for the localized orbitals: cc-pVXZ and aug-cc-pVXZ for X = D, T. A Cholesky orbital initial guess is used in all cases. For the occupied orbitals (left panel), the convergence profiles are similar for all basis sets—near quadratic convergence within 17 iterations. For the virtual orbitals (right panel), we see that the convergence trend deteriorates as the basis set increases. For the largest basis set, aug-cc-pVTZ, convergence is reached in 135 macroiterations. Similar trends in convergence behavior of the virtual orbitals were observed for C_240_ comparing cc-pVDZ and cc-pVTZ in the minimization of the second power of the orbital variance by Høyvik et al.^27^

**Figure 3:**
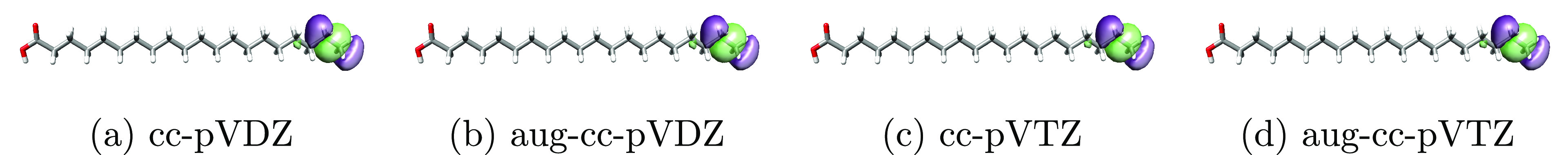
Least local **occupied** Edmiston–Ruedenberg orbitals for arachidic acid, using the cc-pVDZ, aug-cc-pVDZ, cc-pVTZ, and aug-cc-pVTZ basis sets. The orbitals were plotted using an isosurface of 0.01 au.

**Figure 4:**
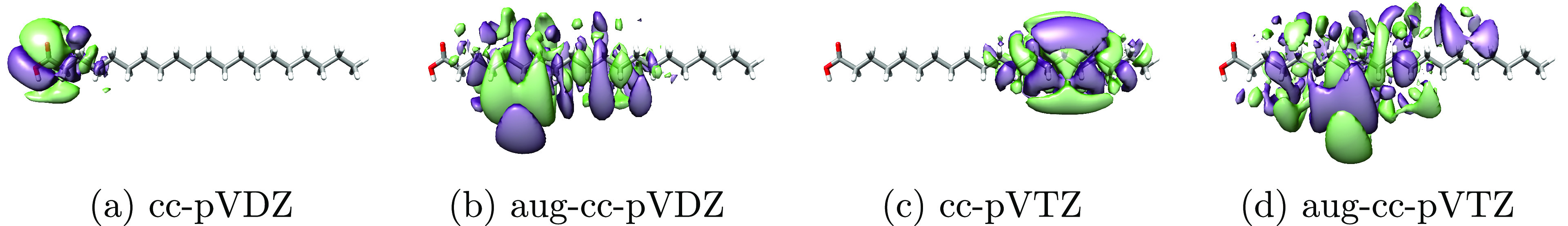
Least local **virtual** Edmiston–Ruedenberg orbitals for arachidic acid, using the cc-pVDZ, aug-cc-pVDZ, cc-pVTZ, and aug-cc-pVTZ basis sets. The orbitals were plotted using an isosurface of 0.01 au.

**Figure 5:**
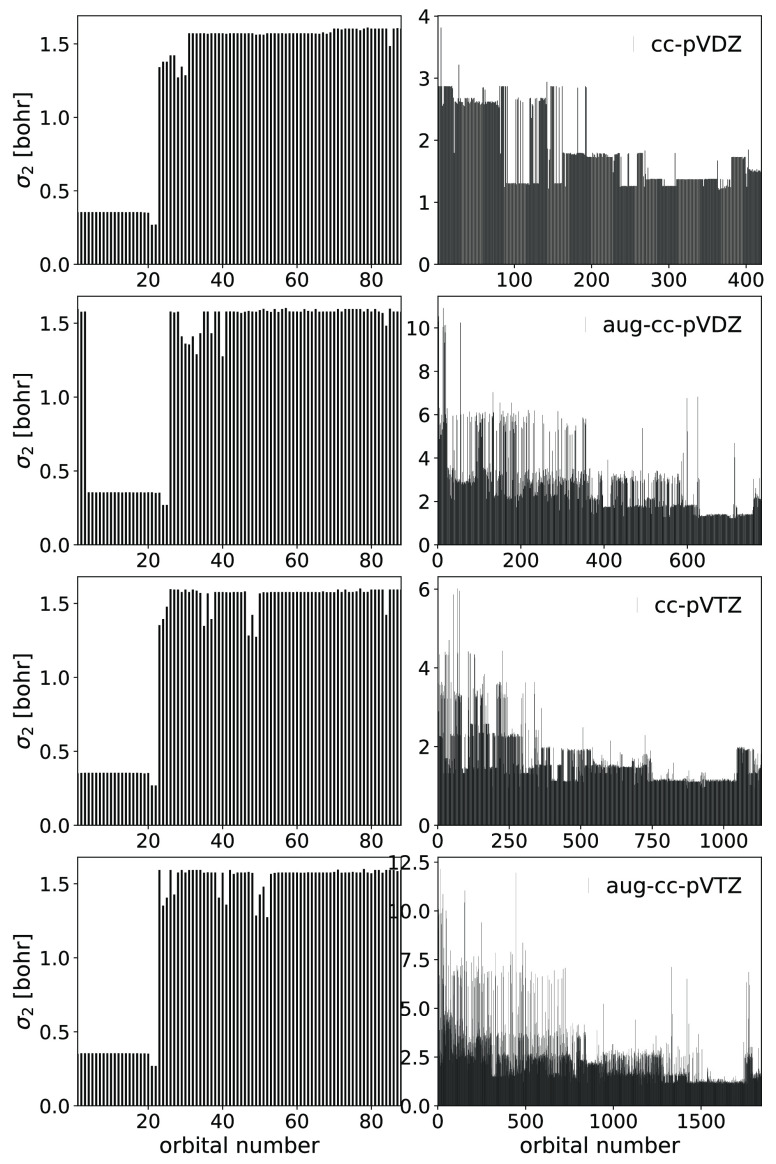
Orbital spreads for Edmiston–Ruedenberg occupied (left panel) and virtual (right panel) orbitals of arachidic acid, obtained in the orbital localization procedure using cc-pVDZ, aug-cc-pVDZ, cc-pVTZ, and aug-cc-pVTZ basis sets.

In Table 1, we present *σ*_2_^*p*^ of the least local occupied and virtual orbitals of arachidic acid for the Cholesky, Foster–Boys, and Edmiston–Ruedenberg Hartree–Fock orbitals. In Figures 3 and 4, we have plotted the least local Edmiston–Ruedenberg orbitals for the cc-pVDZ, aug-cc-VDZ, cc-pVTZ, and aug-cc-pVTZ basis sets using an isosurface of 0.01 au. The full distribution of orbital spreads for these basis sets can be found in Figure 5. As expected, the occupied orbitals are largely unaffected by the change in the basis, whereas the virtual orbitals change significantly. Particularly, the addition of augmenting functions results in several delocalized virtual Edmiston–Ruedenberg orbitals. The results indicate that the Edmiston–Ruedenberg localization function is not well suited for virtual orbital localization. For comparison, localized virtual orbitals of arachidic acid have been reported for several other localization functions in refs (30) and (32); all presented localization functions yielded lower σ_2_^max^ values than the Edmiston–Ruedenberg approach. This is also evident from the σ_2_^max^ values of the calculated Foster–Boys orbitals. The Foster–Boys procedure explicitly minimizes the sum of the second moment orbital spreads and is expected to result in smaller orbital spreads compared to the Edmiston–Ruedenberg approach.

**Table 1:** Values of the Orbital Spread of the Least Local Orbital, σ_2_^max^, of Arachidic Acid Using the Cholesky, Edmiston–Ruedenberg, and Foster–Boys Localization Procedures

		Cholesky		Foster–Boys		Edmiston–Ruedenberg	
basis set	*N*_AO_	occupied	virtual	occupied	virtual	occupied	virtual
cc-pVDZ	508	2.4	8.7	1.6	3.1	1.6	3.8
aug-cc-pVDZ	866	13.7	17.1	1.6	8.4	1.6	10.9
cc-pVTZ	1220	5.9	10.5	1.6	3.6	1.6	6.0
aug-cc-pVTZ	1932	11.6	19.97	1.6	9.4^a^	1.6	12.14

a Unable to converge to 10^–6^ within the given number of iterations, converged to 10^–5^.

In Table 2, we present the wall times used to converge the Hartree–Fock equations (*t*_SCF_) and to localize the occupied and virtual orbitals (*t*_ER,o_ and *t*_ER,v_) of arachidic acid. For all but the smallest basis set, the localization of the occupied orbitals compares favorably to the SCF optimization for this system. For the occupied orbitals, an increase in cost with the basis set size comes from an increase in the number of Cholesky vectors (*N*_*J*_) needed to represent the electron repulsion integrals to the requested accuracy. All contributions are either linear scaling with respect to *N*_*J*_ (**L**, **X**, **Y**, **Z**, and **W**) or nonscaling with respect to *N*_*J*_ (see eq 37).

**Table 2:** Wall Times Used to Converge the Hartree–Fock SCF Equations (*t*_SCF_) and Localize Orbitals (Denoted *t*_ER,o_ for Occupied and *t*_ER,v_ for Virtual Orbitals) for Arachidic Acid

basis set	*N*_AO_	*t*_SCF_	*t*_ER,o_	*t*_ER,v_
cc-pVDZ	508	26.77 s	59.11 s	8.01 min
aug-cc-pVDZ	866	3.06 min	1.18 min	59.14 min
cc-pVTZ	1220	3.05 min	1.13 min	5.19 h
aug-cc-pVTZ	1932	29.78 min	57.68 s	43.05 h

For the virtual orbitals, there is a significant increase in cost as the basis set becomes larger; contrary to the number of occupied orbitals, the number of virtual orbitals increases with the basis set. As seen in Figure 2, more macroiterations are generally needed to reach convergence of the virtual orbitals for larger basis sets.

### Integral Approximation

In this section, we demonstrate the effect of introducing integral approximations through Cholesky decomposition, i.e., by using a decomposition threshold that significantly reduces the number of Cholesky vectors. Significant savings can be obtained in the Edmiston–Ruedenberg localization procedure by such approximations. For circumcoronene, these savings are primarily seen for the virtual space. Savings within the trust-region optimization can come from a reduction in the number of macroiterations or microiterations or from reductions in the number of Cholesky vectors. Reductions (or increases) in the number of iterations arise from the changes in the localization function, due to the approximations in the integrals.

**Figure 6:**
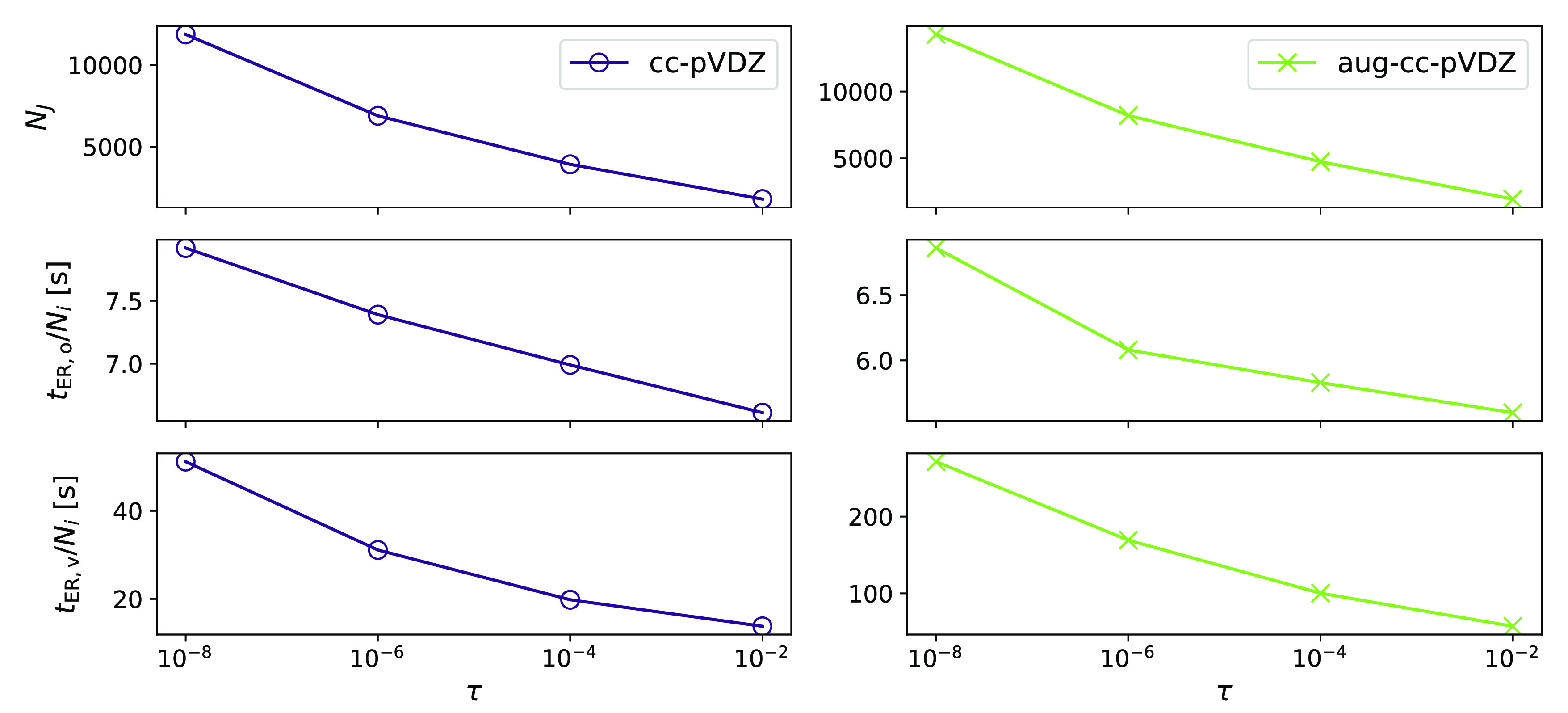
Circumcoronene: the averaged macroiteration time for the occupied (*t*_ER_o__/*N*_*i*_) and virtual (*t*_ER_v__/*N*_*i*_) orbitals for different values of the Cholesky decomposition threshold τ = {10^–8^, 10^–6^, 10^–4^, 10^–2^}. The number of Cholesky vectors, *N*_*J*_, is also plotted as a function of the decomposition threshold.

In Table 3, we present the average wall times per macroiteration needed in the orbital localization for circumcoronene using different Cholesky decomposition thresholds, τ = {10^–8^, 10^–6^, 10^–4^, 10^–2^}, in the cc-pVDZ and aug-cc-pVDZ basis sets. As mentioned, all contributions to the localization function, the corresponding gradient, and the Hessian transformation are either linear scaling or nonscaling with respect to *N*_*J*_. For sizable orbital spaces, we expect the time-limiting steps to be the construction of **L** and **Z**, which occurs once per macroiteration. These steps both scale linearly with *N*_*J*_, and a decrease in cost is expected to be proportional to the decrease in *N*_*J*_. This is seen to be the case for circumcoronene: In Figure 6, we see how the averaged macroiteration time decreases with the number of Cholesky vectors.

**Table 3:** Timings for Localization of Occupied and Virtual Hartree–Fock Orbitals of Circumroronene^a^

			occupied			virtual			
basis	τ	*N*_*J*_	*N*_*i*_	*t*_ER_/*N*_*i*_ (s)	σ_2_^max^	*N*_*i*_	*t*_ER_/*N*_*i*_ (s)	σ_2_^max^	σ_2_^avg^
cc-pVDZ	10^–8^	11870	37	7.92	3.4	96	51.19	3.7	2.2
	10^–6^	6889	33	7.39	3.4	102	31.11	3.6	2.2
	10^–4^	3921	35	6.99	3.4	109	19.8	3.6	2.2
	10^–2^	1794	34	6.61	3.4	62	13.77	4.0	2.2
aug-cc-pVDZ	10^–8^	14278	34	6.86	3.4	146	271.56	13.4	3.3
	10^–6^	8202	34	6.08	3.4	144	169.35	11.6	3.2
	10^–4^	4746	33	5.83	3.4	115	100.27	11.0	3.3
	10^–2^	1942	33	5.60	3.4	115	57.12	14.6	3.3

a τ is the decomposition threshold for the electron repulsion integrals, *N*_*J*_ is the number of Cholesky vectors, *N*_*i*_ is the number of macroiterations in the localization procedure, *t*_ER_ is the time used to localize the orbitals, σ_2_^max^ is the orbital spread of the least local orbital (given in atomic units), and σ_2_^avg^ is the mean of the orbital spreads (given in atomic units). There are 846 atomic orbitals (AOs) in the cc-pVDZ basis and the same number of molecular orbitals (MOs). There are 1404 AOs and 1352 MOs in the aug-cc-pVDZ basis (52 orbitals removed due to linear dependency).

**Figure 7:**
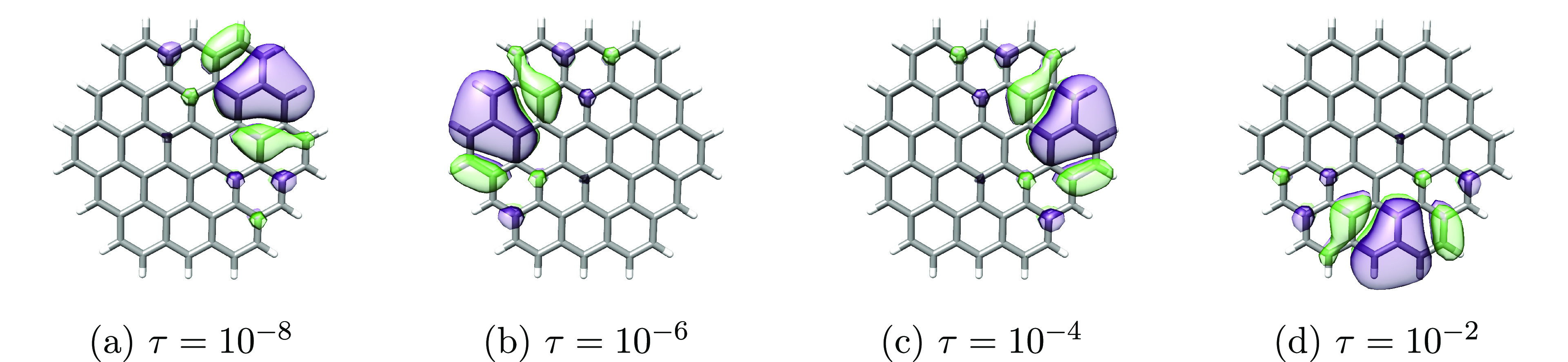
Least local **occupied** orbitals of circumcoronene obtained using different thresholds (τ = {10^–8^, 10^–6^, 10^–4^, 10^–2^}) in the Cholesky decomposition of the electron repulsion integrals and cc-pVDZ basis set. The orbitals were plotted using an isosurface of 0.01 au.

**Figure 8:**
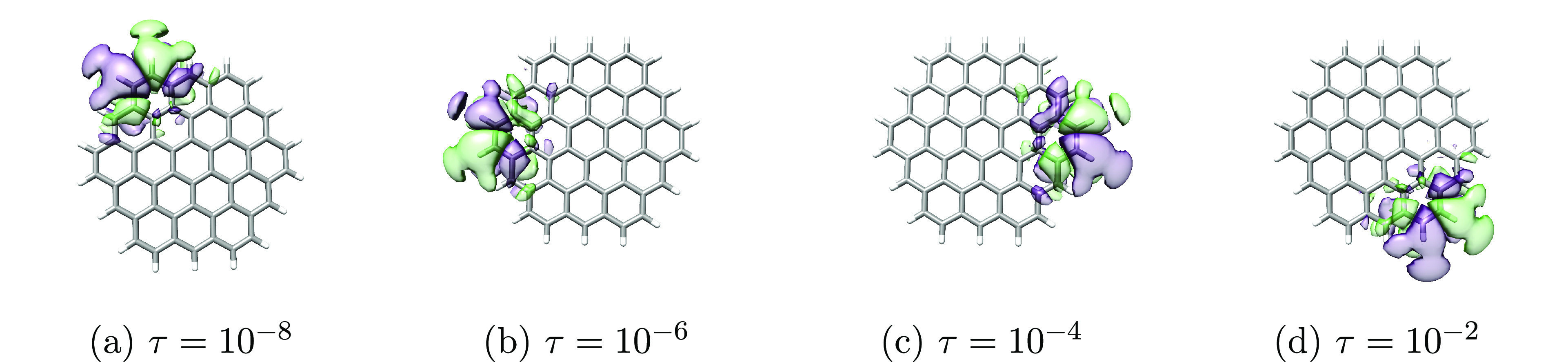
Least local **virtual** orbitals of circumcoronene obtained using different thresholds (τ = {10^–8^, 10^–6^, 10^–4^, 10^–2^}) in the Cholesky decomposition of the electron repulsion integrals and cc-pVDZ basis set. The orbitals were plotted using an isosurface of 0.01 au.

**Figure 9:**
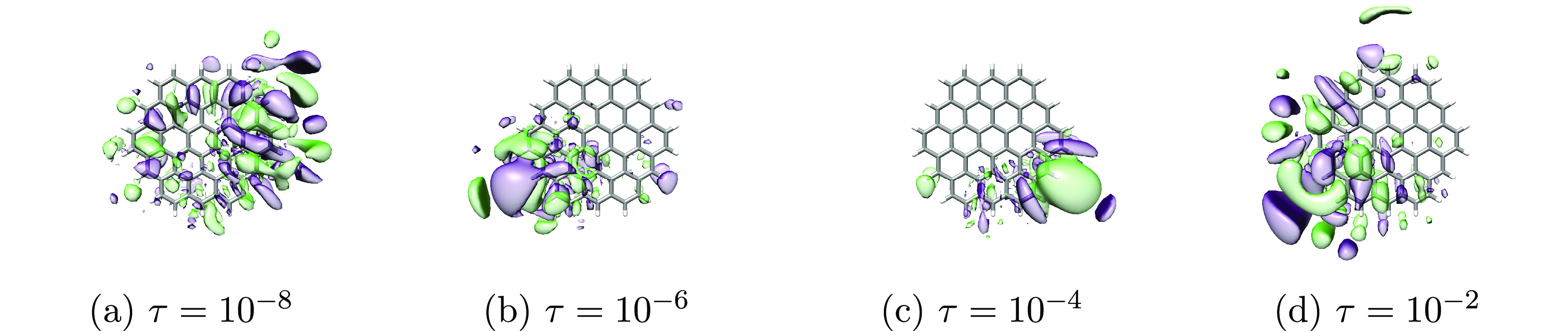
Least local **virtual** orbitals of circumcoronene obtained using different thresholds (τ = {10^–8^, 10^–6^, 10^–4^, 10^–2^}) in the Cholesky decomposition of the electron repulsion integrals and aug-cc-pVDZ basis set. The orbitals were plotted using an isosurface of 0.01 au.

In Table 3, we list the orbital spreads of the least local Edmiston–Ruedenberg orbitals for circumcoronene. For the virtual space, we also present the average orbital spreads. Plots of the least local orbitals (occupied and virtual for cc-pVDZ and virtual for aug-cc-pVDZ) can be found in Figures 7, 8, and 9.

**Figure 10:**
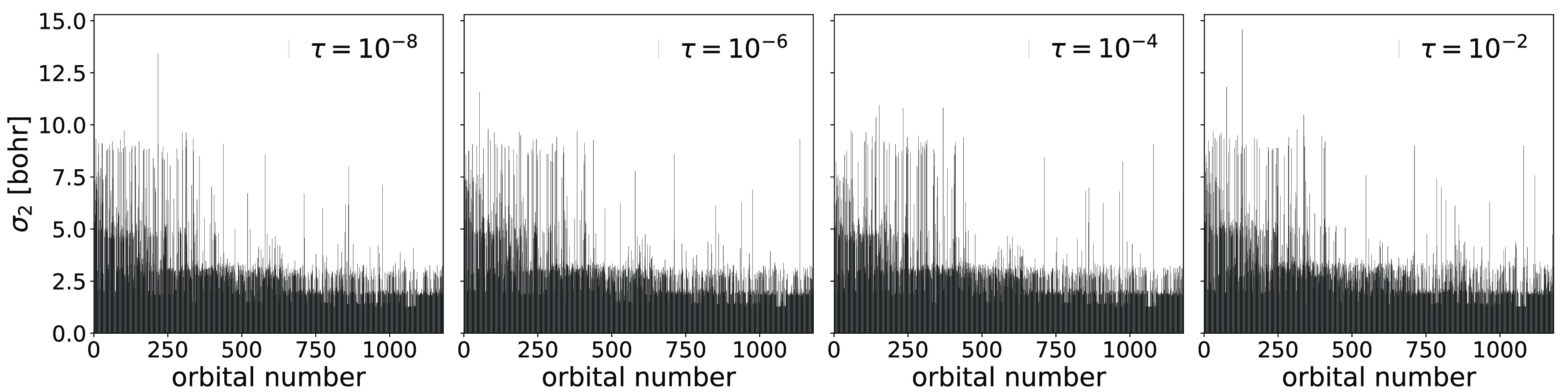
Orbital spreads for Edmiston–Ruedenberg virtual orbitals of circumcoronene, obtained in the orbital localization procedure using different thresholds (τ = {10^–8^, 10^–6^, 10^–4^, 10^–2^}) in the Cholesky decomposition of the electron repulsion integrals and aug-cc-pVDZ basis set.

For cc-pVDZ, the least local orbitals are similar for all decomposition thresholds, and only minor variations in σ_2_^max^ for the virtual space are observed. In contrast, for aug-cc-pVDZ, we see significant variations in the least local virtual orbital for different τ. However, as is evident from the average orbital spread σ_2_^avg^ and the plots of all orbital spreads in Figure 10, the overall locality of the virtual set is largely unaffected by the change in the decomposition threshold. Furthermore, the least local occupied orbitals appear to be outliers, as was also observed by Jansík et al.^26^ for Foster–Boys orbitals. Such outliers could potentially be removed before local post-HF calculations. However, even without these outliers, a large part of the virtual Edmiston–Ruedenberg orbitals of circumcoronene have significant orbital spreads of about 10 au.

**Figure 11:**
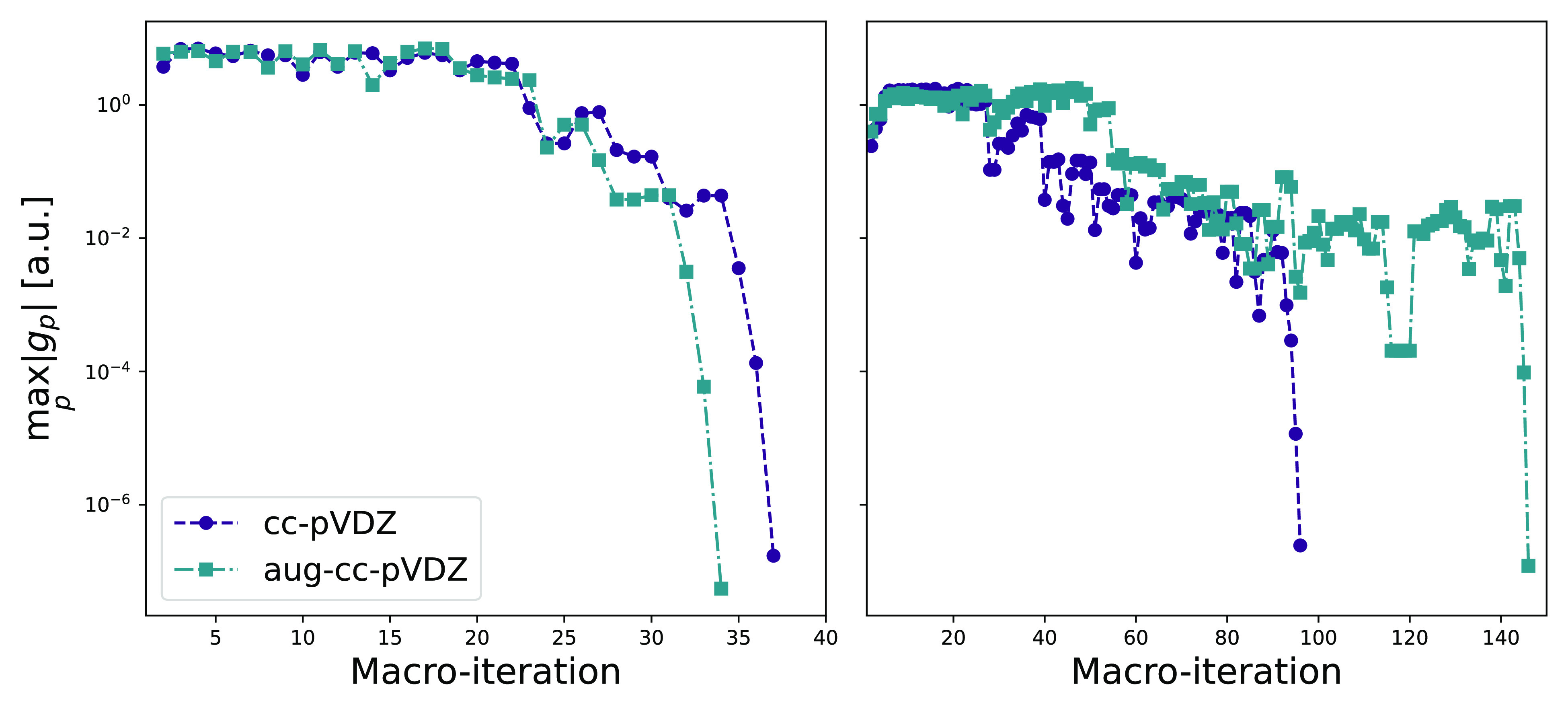
Convergence profiles for the occupied (left panel) and virtual (right panel) orbital localization of circumcoronene. The max gradient norm () is used and is given in atomic units (au).

In Figure 11, we present the convergence profiles of the Edmiston–Ruedenberg localization for circumcoronene, using a decomposition threshold of τ = 10^–8^. The convergence trends are similar to those of arachidic acid (see Figures 1 and 2), with near quadratic convergence.

### Occupied Orbital Localization for Larger Molecular Systems

**Figure 12:**
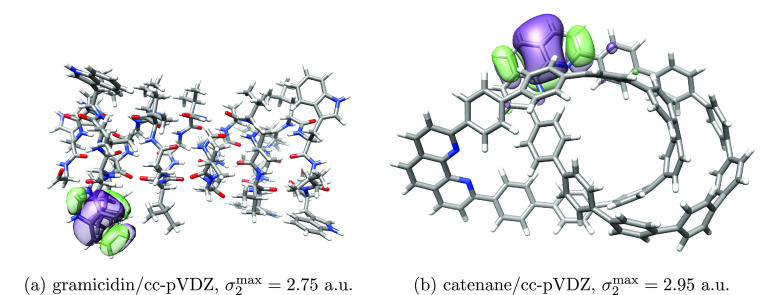
Least local **occupied** orbitals of gramicidin and catenane obtained using the cc-pVDZ basis set, plotted using an isosurface of 0.01 au.

We present occupied orbital localization for two sizable molecular systems: gramicidin and catenane. In Table 4, we present the wall times to localize the occupied orbitals of these systems in the cc-pVDZ basis for τ = {10^–8^, 10^–2^}. We see that the time used to localize the orbitals is shorter than the time used to solve the SCF equations, given an appropriately low Cholesky decomposition threshold. In Figure 12, we have plotted the least local occupied orbitals obtained in the τ = 10^–2^ calculation. In general, the decomposition threshold does not seem to significantly affect the localized occupied orbitals.

**Table 4:** Wall Times for the Edmiston–Ruedenberg Localization of the Occupied Orbitals of Gramicidin and Catenane Using the cc-pVDZ Basis and the Number of Macroiterations Needed to Reach Convergence (*N*_*i*_^ER^)^a^

			initial guess		
system	τ	*t*_SCF_	canonical	Cholesky	Foster–Boys
gramicidin	10^–2^	2.65 h	2.49 h	1.47 h	37.00 min
catenane		21.95 min	6.29 min	3.72 min	3.35 min
gramicidin	10^–8^	2.65 h	14.59 h	8.18 h	3.67 h
catenane		21.95 min	28.44 min	15.62 min	12.41 min

a Different initial guesses for the orbitals are compared: canonical, Cholesky, and Foster–Boys orbitals. The wall times used to converge the SCF equations (*t*_SCF_) are also given.

### Edmiston–Ruedenberg for Local Correlation Methods

**Figure 13:**
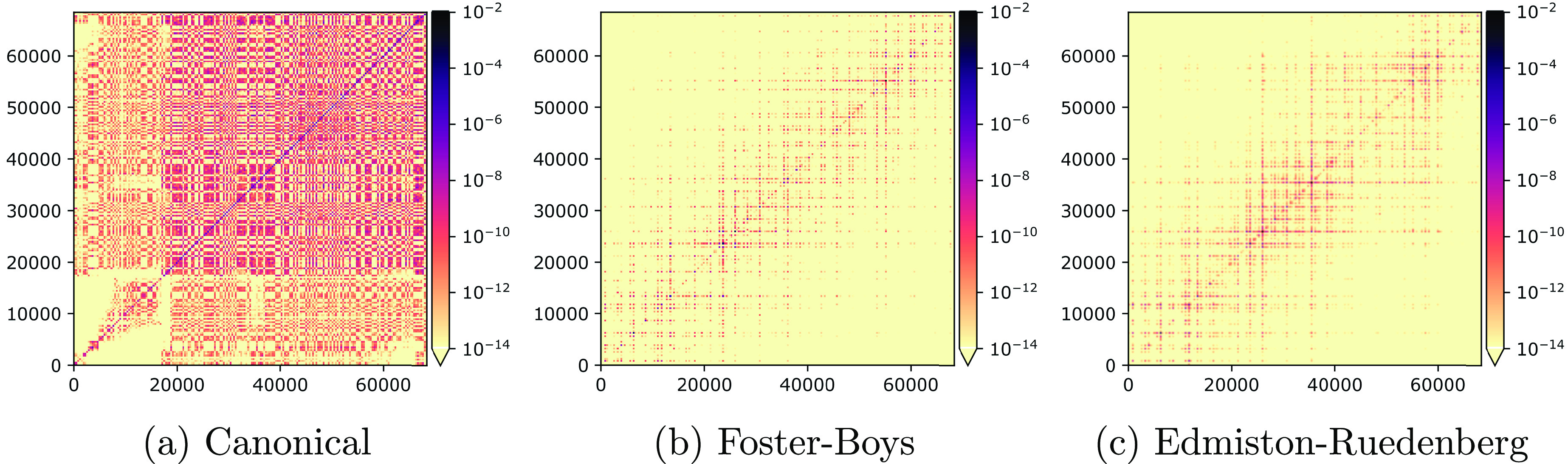
Absolute value of the contributions to the correlation energy *E*_CCSD_^*aibj*^ from the doubles amplitudes *t*_*ij*_^*ab*^ obtained at the CCSD level of theory for arachidic acid/aug-cc-pVDZ, given as a heat map of the *n*_o_*n*_v_ × *n*_o_*n*_v_ matrix.

The foundation for local correlation methods^16,17,34^ is that local operators, such as the electron repulsion operator, will be represented by sparse matrices in the localized MO basis. Hence, the calculation of the correlation energy can be implemented as a linear scaling algorithm. Here, we will consider the potential of Edmiston–Ruedenberg orbitals for use in such local correlation methods. In the (spin-adapted, closed-shell) coupled cluster singles and doubles (CCSD) approach, the correlated wave function is given by
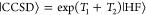
43where  is the Hartree–Fock determinant and
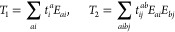
44are the single and double excitation contributions to the cluster operator, given in terms of singlet excitation operators *E*_*pq*_. The CCSD energy is given by
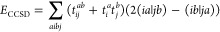
45to which the largest contributions come from the doubles amplitudes:
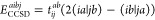
46In Figure 13, we compare the sparsity (or lack thereof) of the *n*_o_*n*_v_ × *n*_o_*n*_v_ matrix with elements *E*_CCSD_^*aibj*^ in the canonical, Foster–Boys, and Edmiston–Ruedenberg bases. In the canonical orbital basis, the matrix is dense, whereas both Foster–Boys and Edmiston–Ruedenberg orbitals result in sparse matrices, demonstrating clearly the local character of the correlation energy. From these preliminary tests, it is difficult to say anything general about the properties of the Edmiston–Ruedenberg orbitals in correlated calculations, except that their performance is comparable to that of Foster–Boys orbitals.

## Concluding Remarks

We have presented a trust-region, second-order optimization of the Edmiston–Rudenberg localization function that can be applied to both the occupied and virtual orbitals. To the best of our knowledge, this is the first successful attempt at localizing the virtual Edmiston–Ruedenberg orbitals. However, some of the resulting virtual Edmiston–Ruedenberg orbitals exhibit large orbital spreads, especially for augmented basis sets. We conclude that other localization functions are more appropriate for virtual orbital localization, particularly those where the localization function explicitly references the orbital spreads.^26,30^

Cholesky decomposition of the electron repulsion integrals reduces the overall scaling of the Edmiston–Ruedenberg localization to . A loose decomposition threshold of τ = 10^–4^ or even τ = 10^–2^ yields good results, with such integral approximations having little effect on the overall locality of the final set of orbitals.

While the virtual orbital localization remains expensive, the occupied orbital localization is comparable to, and in many cases faster than, the cost of the SCF optimization. This is especially the case for larger basis sets. The cost scales linearly with respect to an increase in the basis set for the occupied orbitals.

Further reduction in the scaling may be achieved in an approach similar to that of Subotnik et al.,^13^ where one exploits the locality of an initial guess, i.e., exploiting that, for localized orbitals, *L*_*pq*_^*J*^ is only significant when orbital *p* is close to orbital *q*. The current implementation, however, has the benefit of being simple and independent of the locality of the initial guess while still comparing favorably with the cost of solving the SCF equations.
